# Bulky PP1 analogs exert cellular effects independently from analog-sensitive kinase inhibition

**DOI:** 10.3389/fchem.2026.1812827

**Published:** 2026-04-28

**Authors:** Coralie Gicquel, Sabine Genicot, Arnaud Comte, Pierre Colas

**Affiliations:** 1 Sorbonne Université, CNRS, Laboratoire de Biologie Intégrative des Modèles Marins, LBI2M, Station Biologique de Roscoff, Roscoff, France; 2 ICBMS UMR 5246 CNRS-Université Claude Bernard Lyon 1, Université de Lyon, Villeurbanne, France

**Keywords:** analog-sensitive kinase, chemical genetics, non-specific cellular effects, PP1 analogs, protein kinase inhibition

## Abstract

To circumvent the general lack of selectivity of protein kinase inhibitors, a chemical genetics approach has been developed to allow the selective targeting of engineered kinases by bulky ATP analogs, most of which derived from the pyrazolo[3,4-*d*]pyrimidine 1 (PP1) inhibitor. Although designed to selectively inhibit so-called analog-sensitive (AS) kinases presenting enlarged active sites, bulky PP1 analogs were shown to inhibit a number of wild-type protein kinases *in vitro*. Here, we examine the effects of 5 bulky PP1 analogs on the migration, invasive potential, proliferation, cell cycle, viability and differentiation of non-tumoral and tumoral cell lines that do not express an AS kinase. We show that the three inhibitors that have been employed the most so far (1NA-, 1NM-, 3MB-PP1) produce conspicuous effects on these cellular processes, sometimes at lower concentrations than those often used in AS kinase studies. Our work calls for caution when interpreting some cellular effects obtained with PP1 analogs used at concentrations ≥5 µM or even 2 µM for the least specific of them. Whenever possible, it advocates the use of lower concentrations of 3IB- or 3MSB-PP1 that produce less non-specific effects, as predicted from previously published *in vitro* data.

## Introduction

The study of cellular functions of protein kinases by chemical biology approaches is almost invariably confounded by the imperfect selectivity of small-molecule kinase inhibitors, which often target highly homologous ATP-binding active sites (Elkins et al., 2016; Reinecke et al., 2024). To circumvent this conundrum, a powerful chemical genetic method has been developed (Bishop et al., 2000) and applied to over 150 protein kinases from various organisms over the past 25 years. Its principle consists in mutating the large, so-called gatekeeper amino acid within a kinase active site into a smaller residue, so as to enlarge the ATP-binding pocket. Most so-engineered kinases retain at least partially their catalytic activity and can be inhibited by bulky ATP analogs, most of which are derived from the pyrazolo[3,4-*d*]pyrimidine 1 (PP1) inhibitor (Lopez et al., 2014). Because these bulky inhibitors cannot fit into the large majority of wild-type kinase active sites, their use allows for a selective inhibition of their so-called analog-sensitive (AS) kinase target. However, specificity profiling studies revealed that the molecules first developed (1NA-PP1 and 1NM-PP1) inhibit a few wild-type protein kinases *in vitro* (Anastassiadis et al., 2011; Bain et al., 2007). Moreover, some AS kinases are insensitive to these inhibitors. These findings motivated a medicinal chemistry endeavor that led to the identification of additional active analogs. Among those, 3MB-PP1 (Liu et al., 2009), 3IB-PP1 (Okuzumi et al., 2009) and 3-MSB-PP1 present enhanced potency against a number of AS kinases and an improved, yet still imperfect selectivity (Zhang et al., 2013). Using protein kinase D (PKD) as a case study, a cellular experiment was conducted to support a prediction according to which many of the identified wild-type kinases targeted by PP1 analogs *in vitro* are unlikely to be inhibited in cells at inhibitor concentrations ≤5 µM (Zhang et al., 2013). This result led to the conjecture that the usually employed concentrations of PP1 analogs are not expected to produce conspicuous effects on cells that do not express an AS protein kinase.

Here, we show that 5 bulky PP1 analogs, among which are the 3 most commonly used molecules, affect to varying extents migration, invasiveness, proliferation, cell cycle, viability and differentiation of human cell lines that do not express AS kinases. We observe that many effects are triggered at concentrations lower than those generally used to inhibit AS kinases.

## Results

### PP1 analogs inhibit cell migration

**FIGURE 1:**
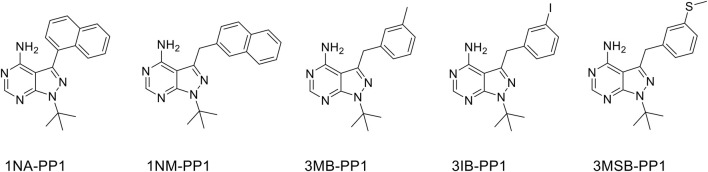
Molecular structures of the tested PP1 analogs. 1NA-, 1NM-, 3MB-, 3IB-PP1 are commercially available. 3MSB-PP1 was synthesized (see *Materials and methods*).

**FIGURE 2:**
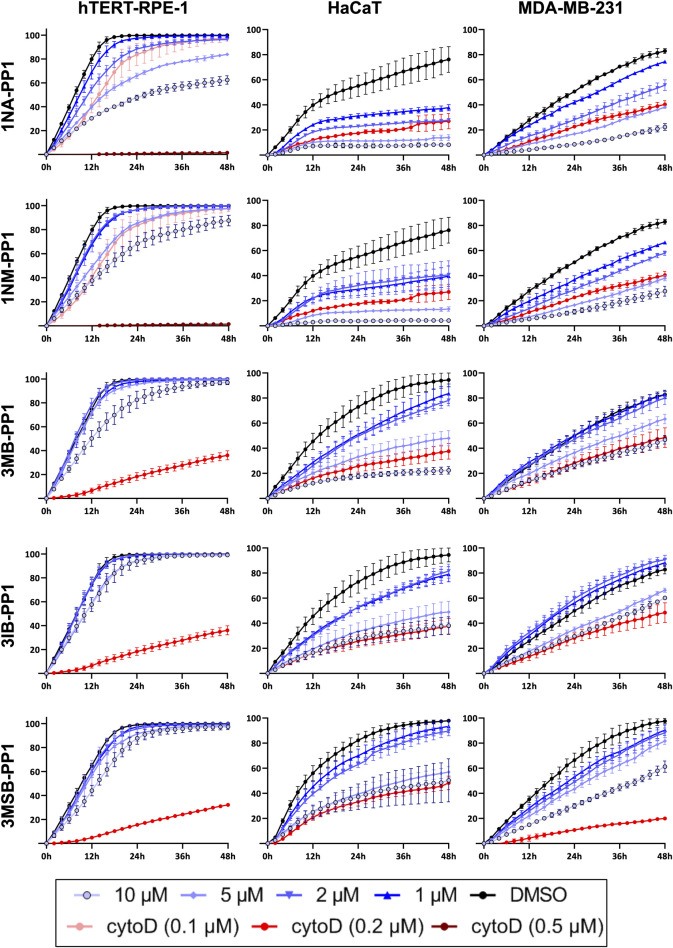
PP1 analogs inhibit cell migration. Cell migration of hTERT-RPE1 (left), HaCaT (middle) and MDA-MB-231 cells (right) was assessed by monitoring wound closure every 2 h over 48 h, in presence of various concentrations of PP1 inhibitors. Cytochalasin D, a potent actin polymerization inhibitor, was used as a reference inhibitor of cell migration (Hayot et al., 2006). Relative wound cellular density was determined in 3 or 4 different wells. Histograms showing relative cellular densities after 12, 24, 48 h of treatments are shown in Supplementary Figure S1.

**FIGURE 3:**
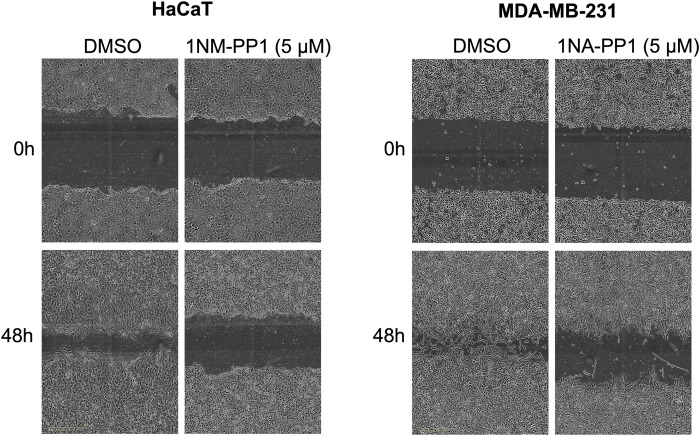
PP1 analogs inhibit cell migration. Examples of HaCaT and MDA-MB-231 cell gaps observed upon wound making and 48 h later, in presence of DMSO or 5 µM of 1NM- and 1NA-PP1, respectively.

In an effort to examine the effects of the inhibition of an AS kinase expressed in a CRISPR/Cas9-edited cell line, we unexpectedly observed conspicuous effects of 1NM-PP1 on wild-type control cells, even at much lower concentrations than those typically used to study AS kinases in cultured cells. This prompted us to test the effects of various concentrations of 5 PP1 analogs (Figure 1) on the migration of hTERT-RPE1 cells (telomerase-immortalized retinal pigmentary epithelial cells), HaCaT cells (immortalized keratinocytes) and MDA-MB-231 cells (derived from a breast adenocarcinoma). To this end, we performed scratches on cell monolayers and we monitored wound healing every 2 h over 48 h (Figure 2). Control DMSO-treated hTERT-RPE1 cells migrated quickly and achieved 100% wound closure in less than 24 h, whereas HaCaT and MDA-MB-231 cells showed a slower migration and achieved variable wound closure 48 h after wound making. 1NM-PP1 and even more so 1NA-PP1 produced a dose-dependent inhibition of the migration of all three cell lines. The inhibition was very pronounced in the slowly migrating HaCaT and MDA-MB-231 cells (Figure 3), readily detected at 2 µM and even 1 µM for the former cell line. 3MB- and 3IB-PP1 produced more limited effects on migration that were only detected at 10 µM in hTERT-RPE1 cells during the first 24 h, and at 5 and 10 µM in MDA-MB-231 cells, throughout the duration of the experiment. HaCaT cell migration was slowed-down by 1µM of 3MB-, 3IB- and 3MSB-PP1 and severely inhibited by 5 and 10 µM of these three molecules (Figure 2; Supplementary Figure S1).

### PP1 analogs inhibit cell invasion

**FIGURE 4:**
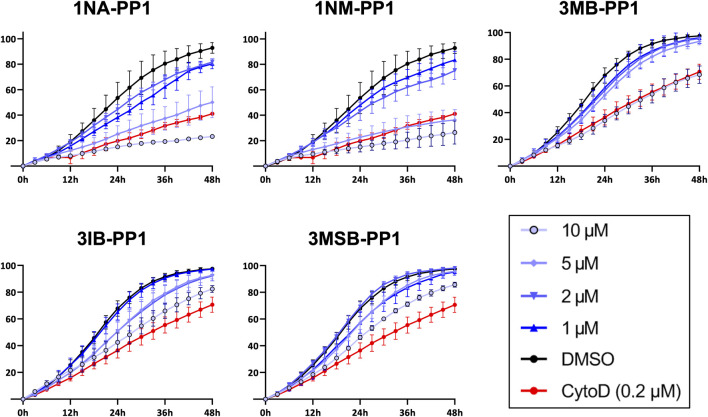
PP1 analogs inhibit cell invasion. MDA-MB-231 cell invasion in presence of a matrigel overlay and different concentrations of PP1 inhibitors was quantified by monitoring cell density within the gaps every 3 h for 48 h after wound making. Cytochalasin D (CytoD) was used as a positive control of inhibition. Experiments were performed in 3 or 4 different wells.

We then tested the effects of PP1 analogs on the highly invasive potential of MDA-MB-231 cells, by performing wound healing assays in presence of a matrigel overlay. All five inhibitors produced dose-dependent inhibitions of cell invasion that mirrored the inhibitions observed on cell migration. Here also, 1NA- and 1NM-PP1 were the most active molecules, since a treatment at 5 µM inhibited cell invasion as much as Cytochalasin D, a potent actin polymerization inhibitor used as control (Figure 4).

### PP1 analogs inhibit cell proliferation

**FIGURE 5:**
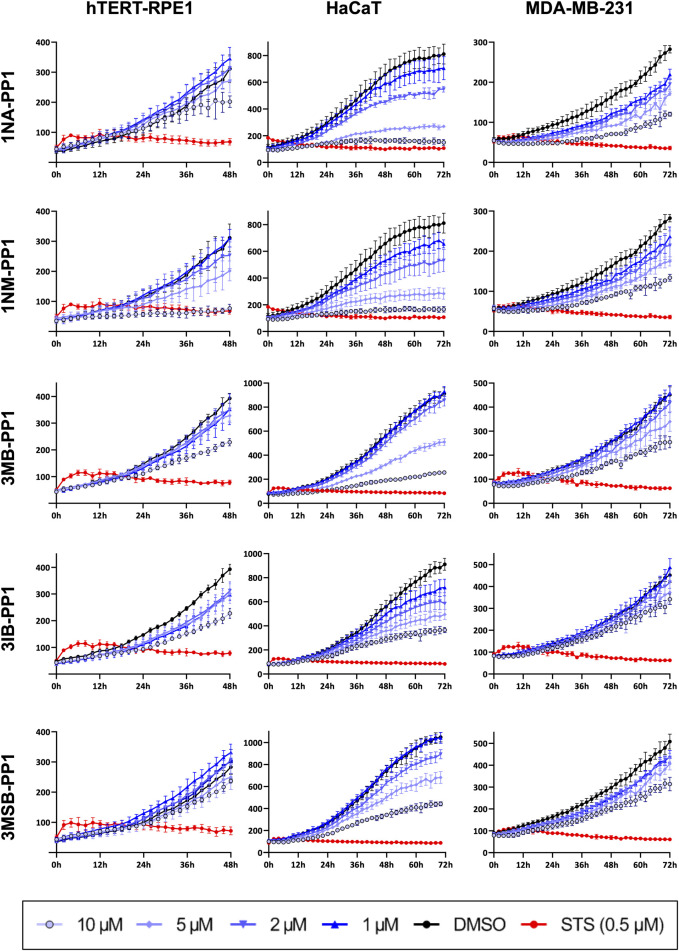
PP1 analogs inhibit cell proliferation. Cell proliferation in presence of different concentrations of PP1 inhibitors was monitored by counting the number of cells every 2 h over 48 or 72 h. Staurosporine (STS) was used as a positive control of inhibition. Experiments were performed in 4 different wells. Histograms showing cell numbers after 12, 24, 48 h or 24, 48, 72 h of treatments are shown in Supplementary Figure S2.

Because wound healing assays are performed in presence of a very low serum concentration, wound closure specifically reflects the ability of cells to migrate and/or invade and does not depend on their ability to proliferate. We thus set out to determine whether PP1 inhibitors also affect cell proliferation by monitoring cell number every 2 h over a period of 48 or 72 h (Figure 5). The proliferation of HaCaT cells was strongly inhibited by 1NM- and 1NA-PP1, with concentration-dependent effects detectable from 1 µM and a total inhibition observed at 10 μM, comparable to that produced by the pan-kinase inhibitor Staurosporine. 3MB-, 3IB- and 3MSB-PP1 also strongly inhibited HaCaT cell proliferation, albeit to a lesser extent. The proliferation of MDA-MB-231 and hTERT-RPE1 cells was generally less sensitive to the PP1 analogs but yet strongly inhibited at 10 μM, or even 5 µM for 1NA- and 1NM-PP1 (Figure 5; Supplementary Figure S2).

### Effects of PP1 analogs on HaCaT cell cycle and viability

**FIGURE 6:**
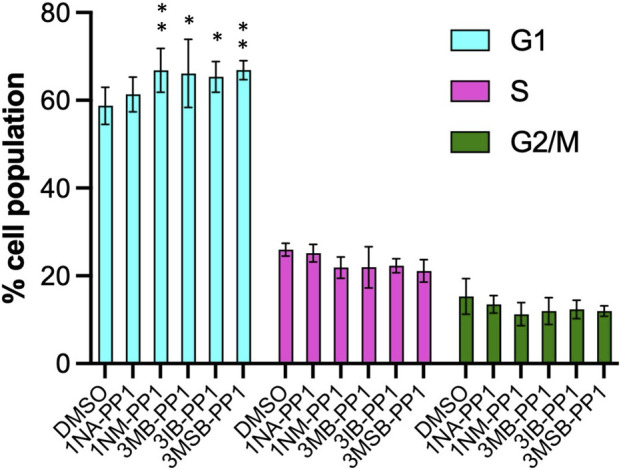
PP1 analogs modify HaCaT cell cycle profile. HaCaT cells were treated with either 0.1% DMSO, 5 µM of 1NA- or 1NM-PP1 or 10 µM of 3MB, 3-IB, or 3MSB-PP1 for 48 h. Cell cycle profiles were determined by flow cytometry after propidium iodide labeling of fixed cells, in four independent experiments. p-values were determined by a two-way ANOVA test followed by Dunnet’s multiple-comparison test comparing each inhibitor to DMSO. Significant differences are labelled with asterisks.

To further characterize the strong inhibition of HaCaT cell proliferation induced by PP1 analogs, we explored their impact on cell cycle profile by flow cytometry. All five inhibitors induced a slight increase of cell population in the G1 phase and slight decreases in S and G2/M phases (Figure 6).

**FIGURE 7:**
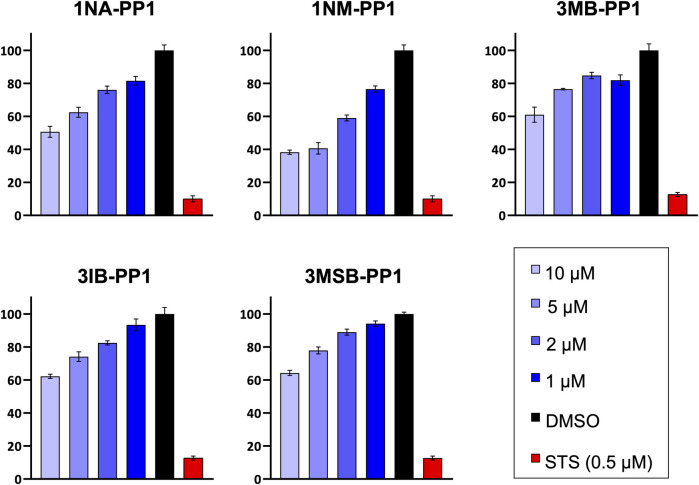
PP1 analogs decrease HaCaT cell viability. Cells were treated with different concentrations of PP1 inhibitors for 72 h and their viability was quantified by an MTS assay. Experiments were performed in quadruplicates.

We then examined the impact of PP1 inhibitors on HaCaT cell viability, as reflected by cell metabolic activity measured by an MTS assay. The PP1 inhibitors produced a pronounced, dose-dependent inhibition of viability (Figure 7). The viability of MDA-MB-231 and hTERT-RPE1 cells was generally much less impacted and it only partially recapitulated the effects of PP1 analogs on cell proliferation (data not shown). Because cells that are subjected to stress or a cell cycle delay undergo important metabolic changes, the overall viability of a cell population does not directly reflect its size, which probably explains this apparent discrepancy.

### Prediction of other biological processes affected by PP1 analogs

**FIGURE 8:**
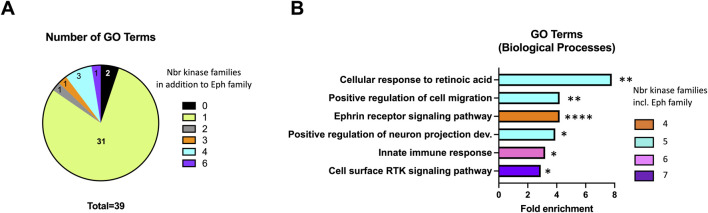
GO terms enrichment analysis within wild-type kinases inhibited by PP1 analogs *in vitro*. **(A)** The 39 biological processes GO terms involving Eph kinases (out of 40 found significantly enriched) were sorted according to the number of additional kinase families they involve. **(B)** Fold enrichment of GO terms that involve at least 4 kinase families (including Eph kinases). Stars indicate statistical significance as determined by p-values.

To predict biological processes that might be affected by PP1 analogs, we performed a gene ontology (GO) term enrichment analysis on the group of 23 wild-type protein kinases reported to be significantly inhibited by these inhibitors *in vitro* (Zhang et al., 2013), using a large subset of the human kinome as reference. Setting the threshold at a minimum of 5 represented kinases per GO term, we found 40 GO biological process terms significantly enriched (Supplementary Table S1). Not surprisingly, since the erythropoietin-producing human hepatocellular receptors kinase family (Eph) accounts for 10 of the 23 inhibited kinases, all but one of the 40 GO terms correspond to biological processes involving Eph kinases. However, only two biological processes involve exclusively Eph kinases while the others involve one to five additional protein kinase families (Figure 8A; Supplementary Table S1). Biological processes that involve 4 to 7 protein kinase families (including the Eph family) and that are thus more likely to be affected by PP1 analogs, are listed in Figure 8B. Besides cell migration shown herein to be strongly inhibited, other important processes such as innate immune response, neuron projection development and/or cellular response to retinoic acid could be impacted by PP1 analogs.

### PP1 analogs accelerate THP-1 macrophage differentiation

**FIGURE 9:**
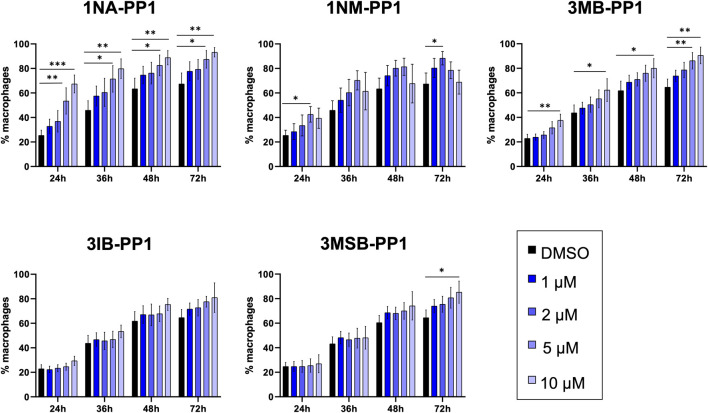
PP1 analogs accelerate THP-1 cell differentiation into macrophages. PMA-induced THP-1 cell differentiation was monitored by counting the number of macrophages every 2 h over 72 h, in presence of different concentrations of PP1 analogs. Three independent experiments were performed. One-way ANOVA statistical analysis was conducted for each time point. Stars indicate statistical significance as determined by p-values.

To obtain a first evidence of the potential impact of PP1 analogs on innate immune response, we examined their effect on the differentiation of monocytes into macrophages. To this end, we treated THP-1 monocytic cells with phorbol 12-myristate 13-acetate (PMA) and various concentrations of PP1 analogs, and we monitored macrophage numbers every 2 h over 72 h. 1NA-, 1NM- and 3MB-PP1 produced a dose-dependent acceleration of macrophage differentiation, observed as soon as 24 h. At the end of the experiment, 72 h after treatment, all three molecules produced a significant increase in the number of differentiated macrophages when used at 10 or even 5 µM for 1NA- and 3MB-PP1, and 2 µM for 1NM-PP1. Higher concentrations of the latter molecule produced a toxic effect, which was observed already 24 h after treatment. Here again, 3IB- and 3MSB-PP1 produced no significant effects, except for the latter molecule at 10 µM after 72 h (Figure 9).

## Discussion

Our results show that bulky PP1 analogs designed to selectively inhibit AS protein kinases exert conspicuous effects on important biological processes in wild-type cells, sometimes at lower concentrations than those most often used. Prior calculations of EC_50_ values using the Cheng-Prusoff equation (Knight and Shokat, 2005; Yung-Chi and Prusoff, 1973) (that takes into account IC_50_ values measured *in vitro*, Km of kinases for ATP, and the average cellular concentration of ATP) predicted that most wild-type kinases inhibited by bulky inhibitors *in vitro* would only be targeted in cells at concentrations higher than 5 µM (Zhang et al., 2013). While this prediction may apply to the wild-type kinases that are the least sensitive to these inhibitors, our results suggest that the most sensitive wild-type kinases are sufficiently inhibited in cells as to trigger significant biological effects, sometimes at low-micromolar concentrations of PP1 analogs.

Prior reports have revealed biological activities of bulky PP1 analogs independently from AS kinase inhibition. However, most reported effects were observed using higher concentrations (≥10 µM) than generally used in AS kinase studies. A treatment of 10 µM 3MB-PP1 of mouse fibroblasts was shown to rescue tunicamycin-induced cellular stress (Maas et al., 2014). Another work reported that 1NA-PP1 treatment (10 and 30 µM) of prostate cancer cell lines inhibits cell invasion and migration. The former effect was attributed to the inhibition of PKDs (Tandon et al., 2013). More recently, even higher concentrations of 1NA-PP1 (over 25 and 55 µM) were reported to inhibit the migration and the invasion of TFK-1 and HuCCT1 cell lines, derived from a bile duct carcinoma and a cholangiocarcinoma, respectively (Lu et al., 2022).

Since PKDs are amongst the most sensitive wild-type kinases to PP1 analogs (Zhang et al., 2013), and considering their widely pleiotropic functions (Zhang et al., 2021), their inhibition is probably involved in at least some of the cellular effects identified herein. Among many examples, PKD small-molecule inhibitors have been shown to inhibit the proliferation of HaCaT cells (Ryvkin et al., 2015). However, the inhibition of additional sensitive kinases likely contributes to AS kinase-unrelated cellular effects. CK1, the epsilon isoform of which being the most sensitive kinase to all five PP1 analogs *in vitro* (Zhang et al., 2013), is involved in the control of the transition between the G1 and S phases of the cell cycle, which could explain the effect of the inhibitors on the cell cycle profile (Dang et al., 2021). Other sensitive kinases are expressed in the 4 cell lines used herein (Supplementary Tables S2, S3) and the inhibition of at least some of them probably contributes to the observed cellular effects.

In addition to the 23 wild-type kinases reported to be inhibited *in vitro* by these molecules (Zhang et al., 2013), hitherto unidentified kinases are probably also targeted. Indeed, off-target inhibitions in cells have been unveiled by a phospho-proteomics comparative analysis between wild-type and CDK12.AS-expressing HeLa cells, treated or not with 10 µM 1NM-PP1 (Bartkowiak et al., 2019). This work revealed 7 common modulated phospho-peptides between CDK12.WT and CDK12.AS cells treated with 1NM-PP1, and 71 phospho-peptides modulated in CDK12.WT-expressing cells upon 1NM-PP1 treatment. Strikingly, none of the potential kinases predicted to phosphorylate the phospho-peptide-corresponding proteins are found amongst the 23 kinases known to be inhibited *in vitro* (Zhang et al., 2013). This somewhat surprising finding illustrates the fact that *in vitro* kinase assays do not always predict inhibitor activities in cells, in which target engagement and inhibition are driven by many factors (Knight and Shokat, 2005). Altogether, GO term-based predictions and experimental findings strongly suggest that PP1 analogs can impact other important biological processes beyond those explored herein.

Our work does not invalidate the vast majority of AS kinase studies reported so far, which include relevant controls involving wild-type kinases. However, it calls for caution when interpreting some cellular effects obtained with PP1 analogs used at concentrations ≥5 µM or even 2 µM for the least specific of them. Whenever possible, it advocates the use of lower concentrations of 3IB- or 3MSB-PP1 that produce less non-specific effects, as predicted from *in vitro* data (Zhang et al., 2013). The orthogonal use of an alternative class of generic bulky inhibitors such as staralogs (Lopez et al., 2013), or even preferably, of a bespoke bulky molecule derived from an inhibitor that targets the cognate WT kinase (Okuzumi et al., 2009) is probably the ideal approach for confidently assigning biological effects to the inhibition of an AS kinase.

## Materials and methods

### Cell cultures

We grew hTERT-RPE1 (telomerase-immortalized human retinal pigment epithelium cells; ATCC CRL-4000), HaCaT (immortalized human keratinocytes; AddexBio T0020001), and MDA-MB-231 (human breast adenocarcinoma cells; ATCC HTB-26) in DMEM medium (Gibco, 61965-059) supplemented with 10% fetal bovine serum (FBS, Gibco, 10270-106) unless otherwise indicated. We grew THP-1 (acute monocytic leukemia cells; ATCC TIB-202) in MEM medium (Gibco, 51200-046) supplemented with 10% FBS, 1% glutamax (Gibco, 35050-061) and 1 mM sodium pyruvate (Gibco, 11360-039). We grew all cell lines and performed all cell incubations described below at 37 °C in a humidified atmosphere containing 5% CO_2_.

### Small-molecule inhibitors

We prepared 10 mM stock solutions of 1NA-PP1 (MedChemExpress, HY-13941), 1NM-PP1 (MedChemExpress, HY-13942), 3MB-PP1 (Aobious, AOB3854), 3IB-PP1(Sigma, 529598) and 3MSB-PP1 in 100% DMSO and we stored aliquots at −20 °C.

### Synthesis of 1-(tert-butyl)-3-(3-(methylthio)benzyl)-1H-pyrazolo[3,4-d]pyrimidin-4-amine (3MSB-PP1)

The synthesis of 3MSB-PP1 was based on the already published original general route (Bishop et al., 1999; Zhang et al., 2013) and experimental details for similar pyrazolopyrimidine compounds (Hanefeld et al., 1996).

Step 1- synthesis of 2-(1-methoxy-2-(3-(methylthio)phenyl) ethylidene)malononitrile: 2-(3-(methylthio)phenyl)acetic acid (830 mg, 4.55 mmol) was suspended in dry CH_2_Cl_2_ (10 mL) under argon and cooled to 0 °C. Oxalyl choride (580 µL, 6.81 mmol, 1.5 eq) was added dropwise and the mixture was stirred for 2 h while returning to room temperature (r.t.). After completion of the reaction, the solvent was removed under vacuum to obtain an oil. In a separate flask, malononitrile (330 mg, 5.01 mmol, 1.1 eq) was dissolved in dry THF (15 mL) under argon, 60% NaH (400 mg, 10.1 mmol, 2.2 eq) was added portionwise and the mixture was stirred for 15 min. The acyl chloride was dissolved in dry THF (10 mL) and added dropwise to the second flask at 0 °C. The reaction was stirred for 2 h while returning to r. t., more NaH was added (182 mg, 1 eq) followed by dimethyl sulfate (647 µL, 6.81 mmol, 1.5 eq), and the mixture was heated to 75 °C for 16 h. The reaction was cooled to r.t., quenched with water and extracted with EtOAc (2 × 30 mL). The extracts were washed with brine, dried over MgSO_4_ and concentrated to a brown oil. Purification by flash chromatography (0%–30% gradient EtOAc: petroleum ether) gave the target compound as a yellow oil (535 mg, 48%). ^1^H NMR (300 MHz, CDCl_3_) δ 7.36–7.27 (m, 1H), 7.22 (d, *J* = 7.7 Hz, 1H), 7.11–7.08 (m, 1H), 6.99 (d, *J* = 7.7 Hz, 1H), 4.05 (s, 3H), 3.97 (s, 2H), 2.50 (s, 3H).
Step 2- synthesis of 5-amino-1-(tert-butyl)-3-(3-(methylthio)benzyl)-1H-pyrazole-4-carbonitrile: 2-(1-methoxy-2-(3-(methylthio)phenyl) ethylidene)malononitrile (530 mg, 2.17 mmol) was dissolved in ethanol (15 mL) with tert-butylhydrazine hydrochloride (170 mg, 2.17 mmol, 1 eq) and Et_3_N (300 µL, 2.17 mmol, 1 eq). The mixture was refluxed under argon for 3.5 h and cooled to r. t. The reaction was worked up by addition of water and extraction with EtOAc (2 × 30 mL). The extracts were washed with brine, dried over MgSO_4_ and concentrated to a yellow oil. Purification by flash chromatography (50% CH_2_Cl_2_: petroleum ether to 100% CH_2_Cl_2_ then 0%–5% gradient EtOAc:CH_2_Cl_2_) gave the target compound as a yellow oil (390 mg, 60%). ^1^H NMR (300 MHz, CDCl_3_) δ 7.24–7.17 (m, 2H), 7.12–7.06 (m, 2H), 4.24 (s, 2H), 3.85 (s, 2H), 2.47 (s, 3H), 1.61 (s, 9H) : 13C NMR (126 MHz, CDCl3) δ 150.4, 150.0, 139.0, 138.6, 129.0, 127.0, 125.8, 125.0, 114.7, 59.3, 34.1, 29.3, 15.9. HRMS (ESI): m/z calculated for C16H20N4S [M+H]+ 301.1481, found 301.1483.
Step 3- synthesis of 1-(tert-butyl)-3-(3-(methylthio)benzyl)-1H-pyrazolo[3,4-d]pyrimidin-4-amine: 5-amino-1-(tert-butyl)-3-(3-(methylthio)benzyl)-1H-pyrazole-4-carbonitrile (340 mg, 1.13 mmol) was suspended in formamide (4 mL) and refluxed under argon for 6 h. The reaction was cooled to r. t, worked up by addition of water and extracted with EtOAc (2 × 20 mL). The extracts were washed with brine, dried over MgSO_4_ and concentrated to a brown oil. Purification by flash chromatography (0%–50% gradient EtOAc:CH_2_Cl_2_) gave the final compound 3MSB-PP1 as a light yellow solid (202 mg, 55%). ^1^H NMR (300 MHz, CDCl_3_) δ 8.27 (s, 1H), 7.22–7.22 (m, 1H), 7.15–7.10 (m, 2H), 6.95 (d, *J* = 7.5 Hz, 1H), 4.87 (s, 2H), 4.27 (s, 2H), 2.44 (s, 3H), 1.81 (s, 9H) 13C NMR (126 MHz, CDCl3) δ 157.7, 154.7, 140.6, 140.0, 139.1, 129.8, 126.4, 125.2, 125.1, 100.8, 60.1, 35.2, 29.3, 15.7. HRMS (ESI): m/z calculated for C_17_H_21_N_5_S [M+H]^+^ 328.1590, found 328.1594. Raw NMR data of the two intermediates and the final molecule are provided in Supplementary File 1.

### Cell migration assays

We seeded hTERT-RPE1 (2 × 10^4^ cells/well), MDA-MB231 (3.5 × 10^4^ cells/well), or HaCaT (4 × 10^4^ cells/well) in 96 well culture plates (Sartorius, BA-04856) and grew them for about 24 h until they reached confluence. We changed the medium with DMEM 1% FBS for 2 h. We performed a median wound on the cell layer in each well using a Woundmaker device (Sartorius, BA-04858). We treated cells with 1, 2, 5 or 10 µM of PP1 analogs or 0.1% DMSO or different concentrations of Cytochalasin D (Sigma, C2618) diluted in DMEM 1% FBS. We monitored wound closures every 2 h over 48 h using a cell live imager (Sartorius, Incucyte SX5).

### Cell invasion assays

We coated 96 well plates (Sartorius, BA-04856) with 100 μg/mL matrigel (Corning, 354234). We incubated the plate overnight, discarded the matrigel and seeded 3.5 × 10^4^ MDA-MB-231 cells/well. We incubated for 4 h and performed a median wound on the cell layer in each well as described above. We washed cells twice with DMEM medium and we added 50 µL/well of 8 mg/mL matrigel containing different concentrations of PP1 analogs or 0.1% DMSO or 200 nM Cytochalasin D. We let polymerize 30 min in the incubator and we added PP1 analogs or DMSO or Cytochalasin D prepared in DMEM 1% FBS. We monitored wound invasion every 3 h over 48 h using the cell live imager.

### Cell proliferation assays

We seeded hTERT-RPE1 (2.10^3^ cells/well), HaCaT (5.10^3^ cells/well) or MDA-MB-231(5.10^3^ cells/well) in 96 well plates (CytoOne, CC7682-7596) and grew them for 24 h. We treated cells with PP1 analogs or 0.1% DMSO or 0.5 µM Staurosporine (Sigma, S5921) prepared in DMEM 10% FBS. We monitored the number of cells every 2 h over 48 h (hTERT-RPE1 cells) or 72 h (HaCaT and MDA-MB-231 cells) using the cell live imager and the Incucyte AI Cell Health Analysis Software Module (Sartorius, BA-04871).

### Cell viability assays

At the end of the above-described cell proliferation assays, we determined cell viability by adding 20 µL/well of CellTiter 96 AQueous One Solution Reagent (Promega, G3581) and incubated for 3 h at 37 °C. To assess the amount of soluble formazan produced by cellular reduction of MTS, we used a Biotek microplate reader (Agilent) to measure the absorbance at 490 nm, with a correction at 630 nm to subtract background contributed by excess cell debris and other nonspecific absorbance. We determined cell survival percentages using the following equation:

$$\text{Survival fraction}=100x\left(\text{mean OD in test wells }-\text{ mean OD in cell}-\text{free wells}\right)/\left(\text{mean OD in control wells }-\text{ mean OD in cell}-\text{free wells}\right).$$

### Determination of cell cycle profiles

We determined the cell cycle distribution of HaCaT cells by staining cells with propidium iodide (PI) and quantifying DNA content by flow cytometry. In brief, we seeded HaCaT cells (1.1 × 10^4^ cells/cm^2^) in T75 flasks (CytoOne, CC7682-4175) and grew them for 24 h. We then treated the cells either with 0.1% DMSO or with 10 µM 3IB-, 3MB- or 3MSB-PP1 analogs. Due to their drastic effect on cell proliferation, we used only 5 µM of 1NA- and 1NM-PP1. 48 h after treatment, we harvested the cells and fixed them for at least 2 h in 70% ice-cold ethanol. To perform PI labelling, we incubated 7.5 × 10^5^ fixed cells for 30 min at 37 °C in 1 mL PBS containing 40 µg PI (Invitrogen, P3566) and 10 µg RNAse A (Fluka, 83831). We loaded the cells into an Attune NxT flow cytometer (Thermofisher) at a flow rate of 25 μL/min and set the voltages as follows: FSC: 94; SSC: 285 and BL2: 380. We analyzed the data using Kaluza analysis flow cytometry software v.2.2.1 (Beckman Coulter).

### GO term analysis

We extracted Uniprot accession identifiers for the 23 protein kinases reported to be significantly inhibited by bulky PP1 analogs *in vitro* (Zhang et al., 2013), taking into account the longer of the two Lyn protein splice variants (P07948). We used the Uniprot accession identifiers of 303 serine/threonine (Johnson et al., 2023) and 93 tyrosine (Yaron-Barir et al., 2024) kinases recently listed. We performed a GO term enrichment analysis by running the 23 sensitive kinases against this large subset of the human kinome, using the DAVID bioinformatics webserver (Huang et al., 2009; Sherman et al., 2022). We only took into account biological processes GO terms (GOTERM_BP_DIRECT) found in at least 5 inhibited kinases (out of 23), with p values ≤0.05.

### Macrophage differentiation assays

We seeded THP-1 cells (3.5 × 10^4^ cells/well) in 96 well plates and we induced differentiation with 50 nM phorbol 12-myristate-13-acetate (Sigma, P1585) in presence of either 0.1% DMSO or 1, 2, 5 or 10 µM of PP1 analogs. We monitored the differentiation into macrophages every 2 h over 72 h using the cell live imager and the Incucyte AI Cell Health Analysis Software Module. We determined the percentage of macrophages using the Incucyte Advanced Label-Free Classification Analysis Software Module.

## Data Availability

The original contributions presented in the study are included in the article/Supplementary Material; further inquiries can be directed to the corresponding author.

## Supplementary material

SUPPLEMENTARY TABLE S1 GO terms enrichment analysis within wild-type kinases inhibited by PP1 analogs *in vitro*. The selection of the 40 enriched GO terms is explained in the *Materials and methods* section. Number (count) and percentage (%) of kinases involved in the biological processes designated by the GO terms, among the list of 23 wild-type kinases inhibited by PP1 analogs *in vitro*. Eph kinase names are in grey to highlight the names of the other kinases. The last column indicates the number of protein kinase families that are represented for each GO term (including the Eph kinase family).

SUPPLEMENTARY TABLE S2 Protein expression levels in MDA-MB-231 and THP-1 cells of wild-type kinases inhibited by PP1 analogs *in vitro*. We extracted protein expression levels in MDA-MB231 and THP-1 cells from the ProCan-DepMapSanger datasets (Gonçalves et al., 2022). z-score normalization sets the mean expression level in all analyzed cell lines at 0. Positive and negative z-scores indicate expression values above and under the mean, respectively.

SUPPLEMENTARY TABLE S3 RNA expression levels in hTERT-RPE1, HaCaT and THP-1 cells of wild-type kinases inhibited by PP1 analogs. We extracted RNA expression levels in hTERT-RPE1, HaCaT and THP-1 cells from the Human Protein Atlas website (https://www.proteinatlas.org/). nTPM (normalized transcripts per million) values are indicated.

SUPPLEMENTARY FIGURE S1 Quantification of cell migration 12, 24 and 48 h after wound making. Relative wound densities (expressed as percentages on Y-axis) within the gaps 12, 24 and 48 h after wound making were extracted from the real-time monitoring of cell migration shown in Figure 2.

SUPPLEMENTARY FIGURE S2 Cell counts after different treatment durations with PP1 analogs. Cell numbers were extracted from the real-time monitoring of cell proliferation shown in Figure 5.
