# Supplementary figures and images for: Bulky PP1 analogs exert cellular effects independently from analog-sensitive kinase inhibition
Source: Front Chem. 2026 Apr 28;14:1812827. doi: 10.3389/fchem.2026.1812827 (PMC13161952; doi:10.3389/fchem.2026.1812827)

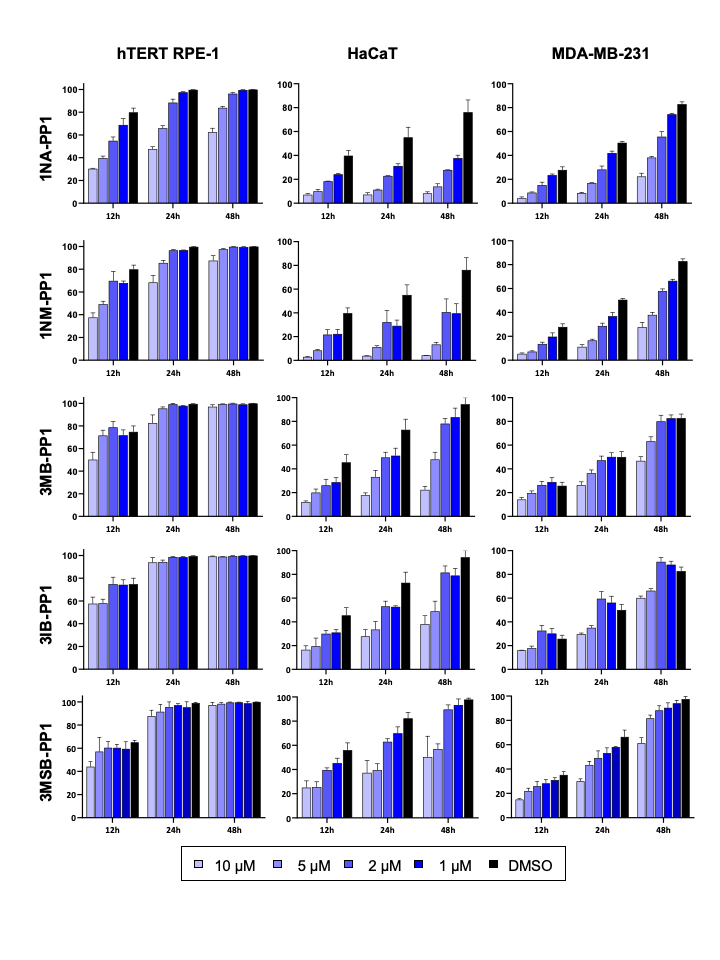

Supplement: Supplementary file 2 [file Image1.tiff]

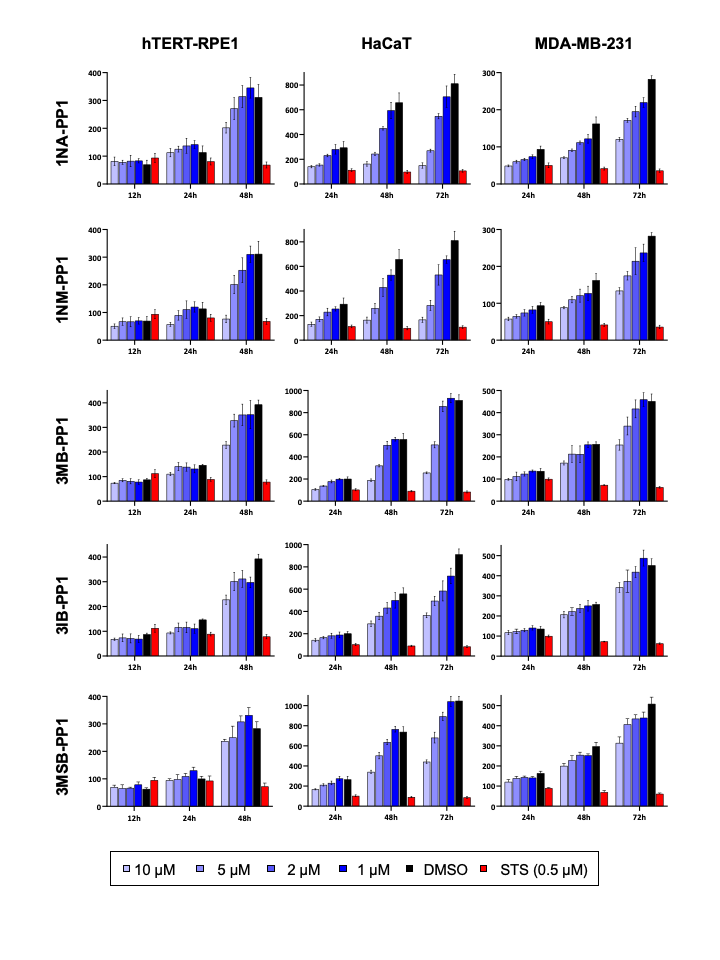

Supplement: Supplementary file 6 [file Image2.tiff]

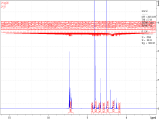

Supplement: Supplementary file 7 [file Supplementaryfile1.zip › NMR_rawdata/intermediate 1/1H/pdata/1/thumb.png]
