# Supplementary material for: Bulky PP1 analogs exert cellular effects independently from analog-sensitive kinase inhibition
Source: Front Chem. 2026 Apr 28;14:1812827. doi: 10.3389/fchem.2026.1812827 (PMC13161952; doi:10.3389/fchem.2026.1812827)
Supplement: Supplementary file 4 [file Image3.pdf]

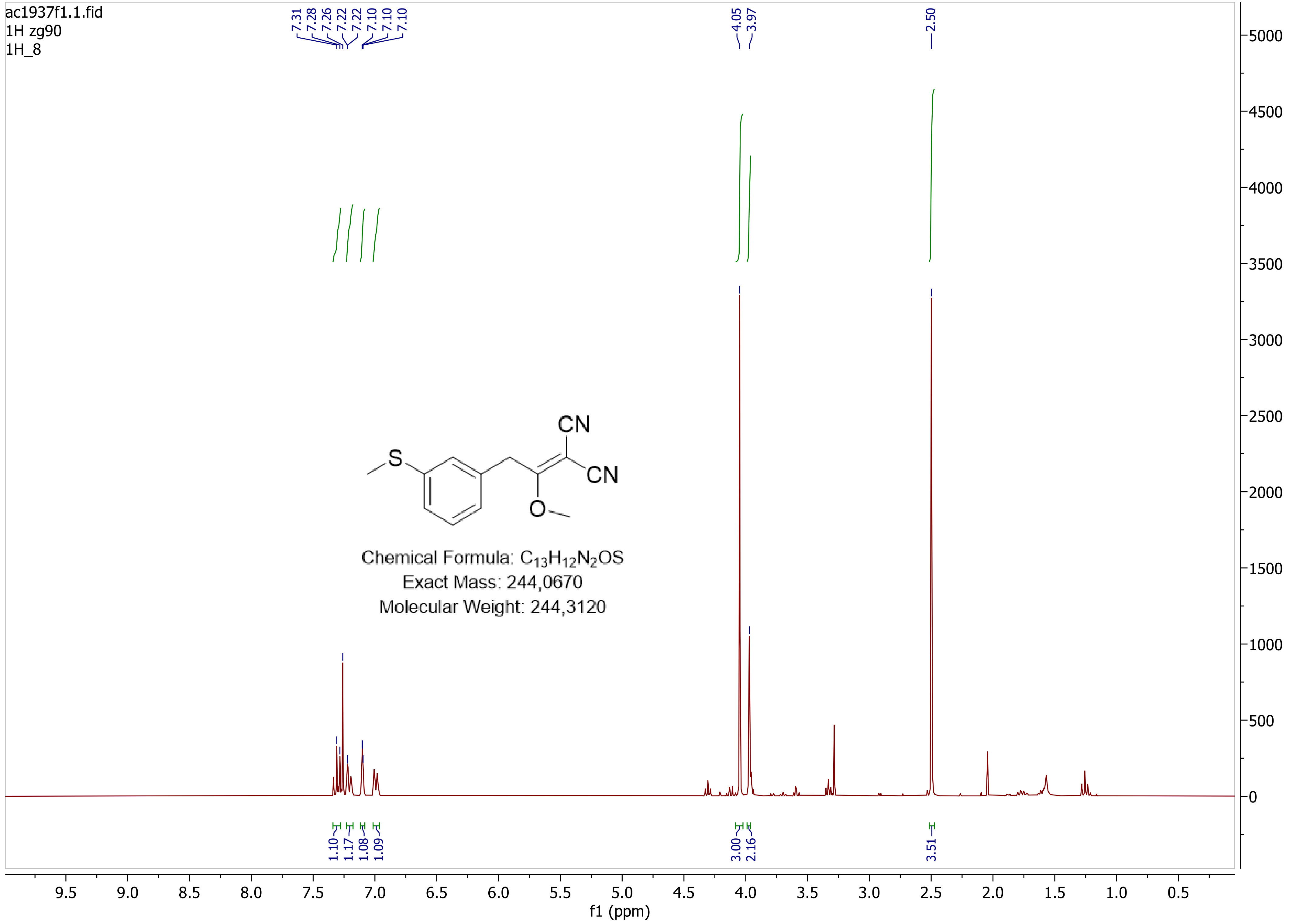
ac1937f1.1.fid
1H zg90
1H_8
7.31
7.28
7.26
7.22
7.22
7.10
7.10
7.10
4.05
3.97
2.50
CN
S
CN
O
Chemical Formula: $C_{13}H_{12}N_2OS$
Exact Mass: 244,0670
Molecular Weight: 244,3120
1.10
1.17
1.08
1.09
3.00
2.16
3.51
9.5 9.0 8.5 8.0 7.5 7.0 6.5 6.0 5.5 5.0 4.5 4.0 3.5 3.0 2.5 2.0 1.5 1.0 0.5
f1 (ppm)
5000 4500 4000 3500 3000 2500 2000 1500 1000 500 0

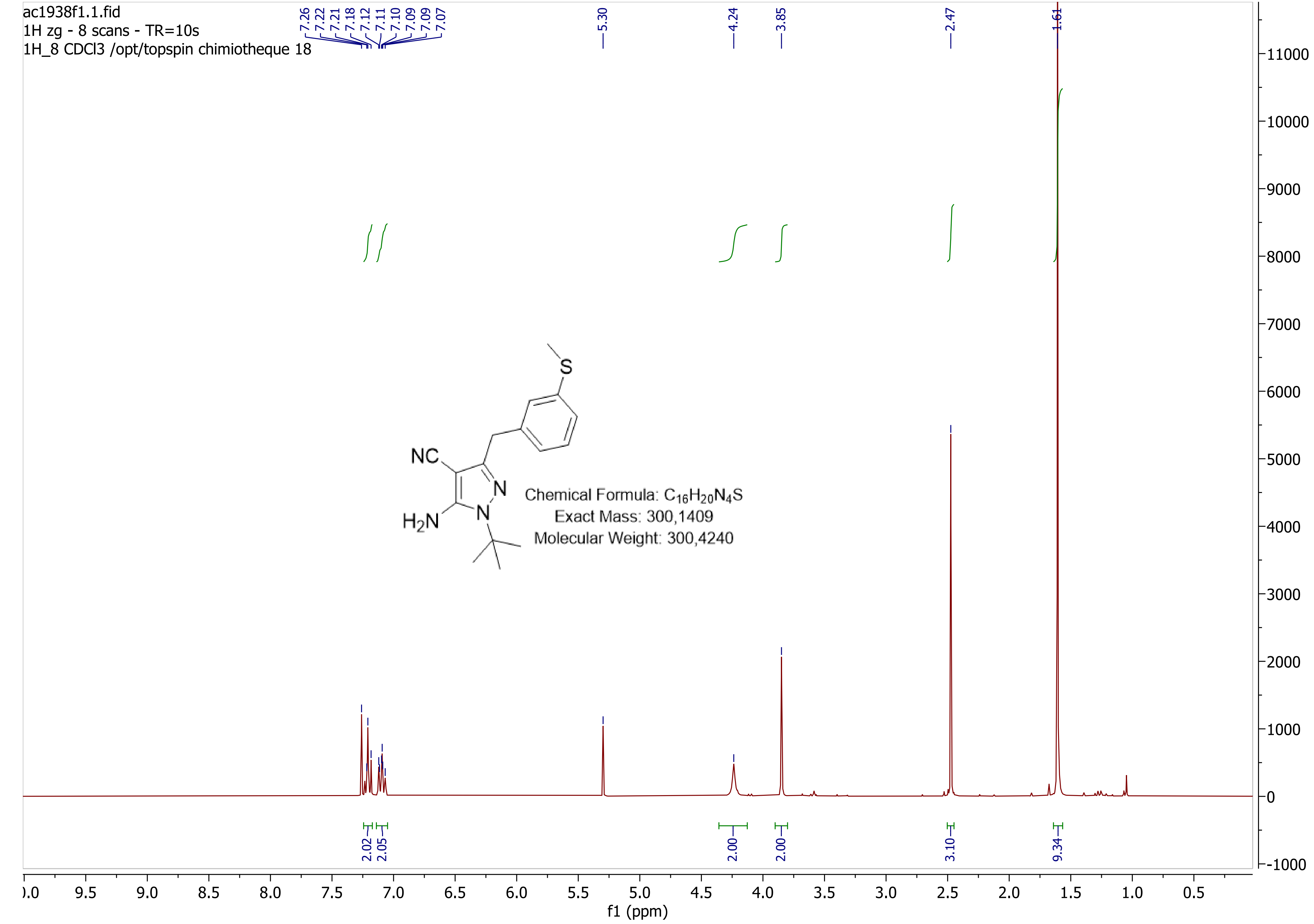
ac1938f1.1.fid
1H zg - 8 scans - TR=10s
1H_8 CDCl3 /opt/topspin chimiotheque 18
7.26
7.22
7.21
7.18
7.12
7.11
7.10
7.09
7.09
7.07
5.30
4.24
3.85
2.47
1.61
NC
N
N
H_2N
S
Chemical Formula: $C_{16}H_{20}N_4S$
Exact Mass: 300,1409
Molecular Weight: 300,4240
2.02
2.05
2.00
2.00
3.10
9.34
f1 (ppm)

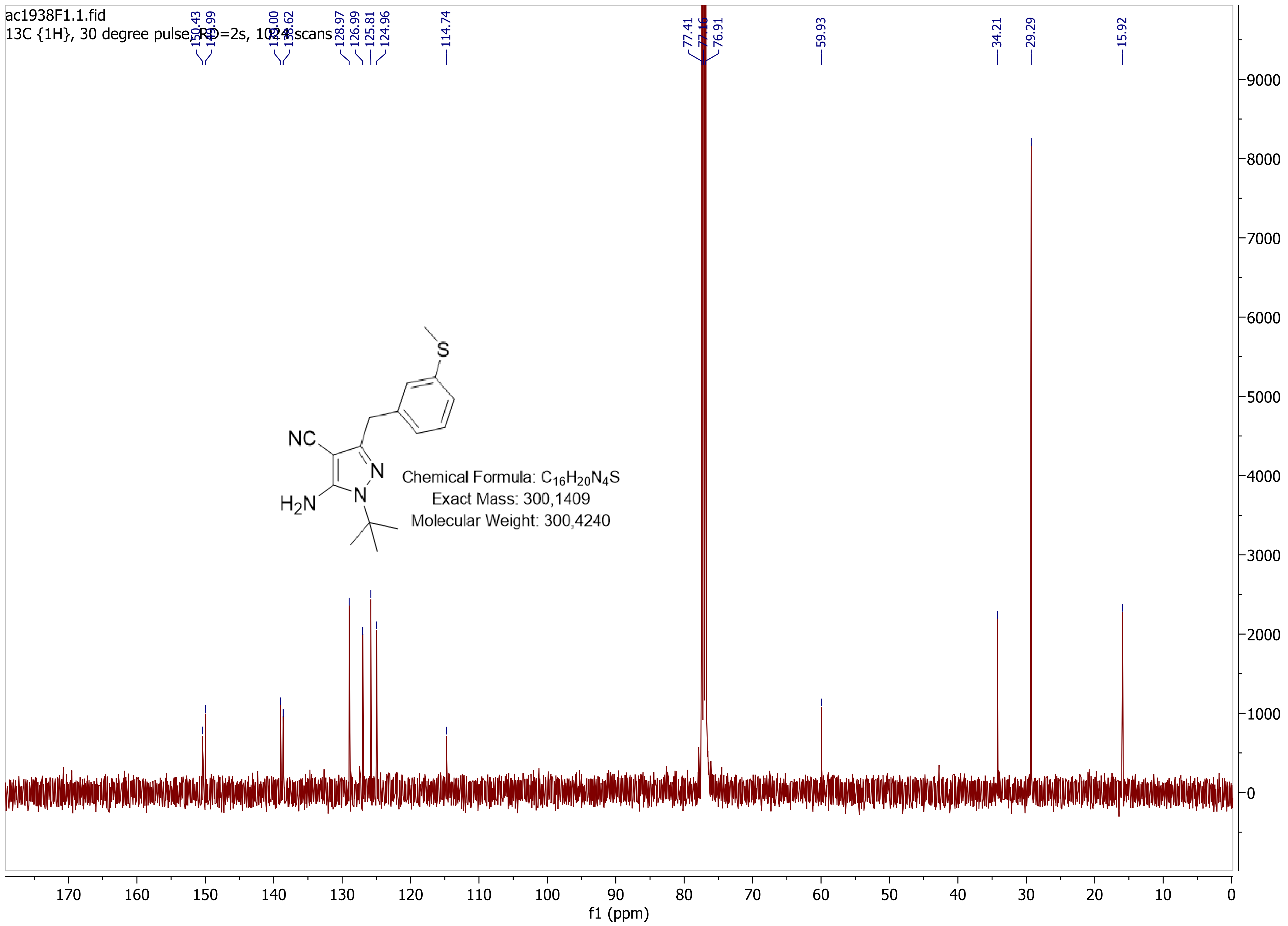
ac1938F1.1.fid
13C {1H}, 30 degree pulse, RD=2s, 1024 scans
150.43
149.99
138.62
128.97
126.99
125.81
124.96
114.74
77.41
77.16
76.91
59.93
34.21
29.29
15.92
NC
N
N
H_2N
S
Chemical Formula: $C_{16}H_{20}N_4S$
Exact Mass: 300,1409
Molecular Weight: 300,4240
9000
8000
7000
6000
5000
4000
3000
2000
1000
0
170
160
150
140
130
120
110
100
90
80
70
60
50
40
30
20
10
0
f1 (ppm)

ac1939f1.1.fid
1H zg - 8 scans - TR=10s
1H_8 CDCl3 /opt/topspin chimiotheque 22

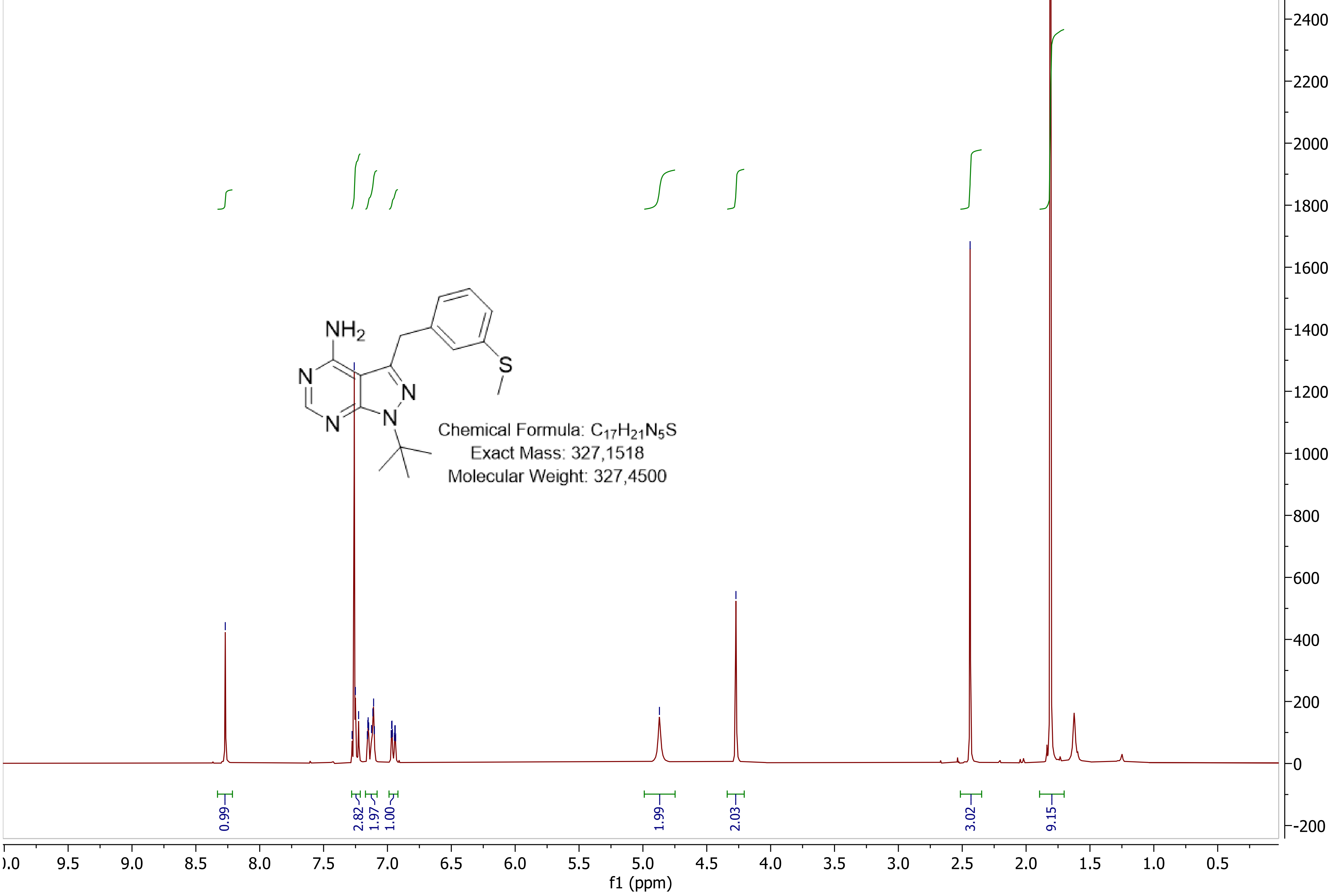

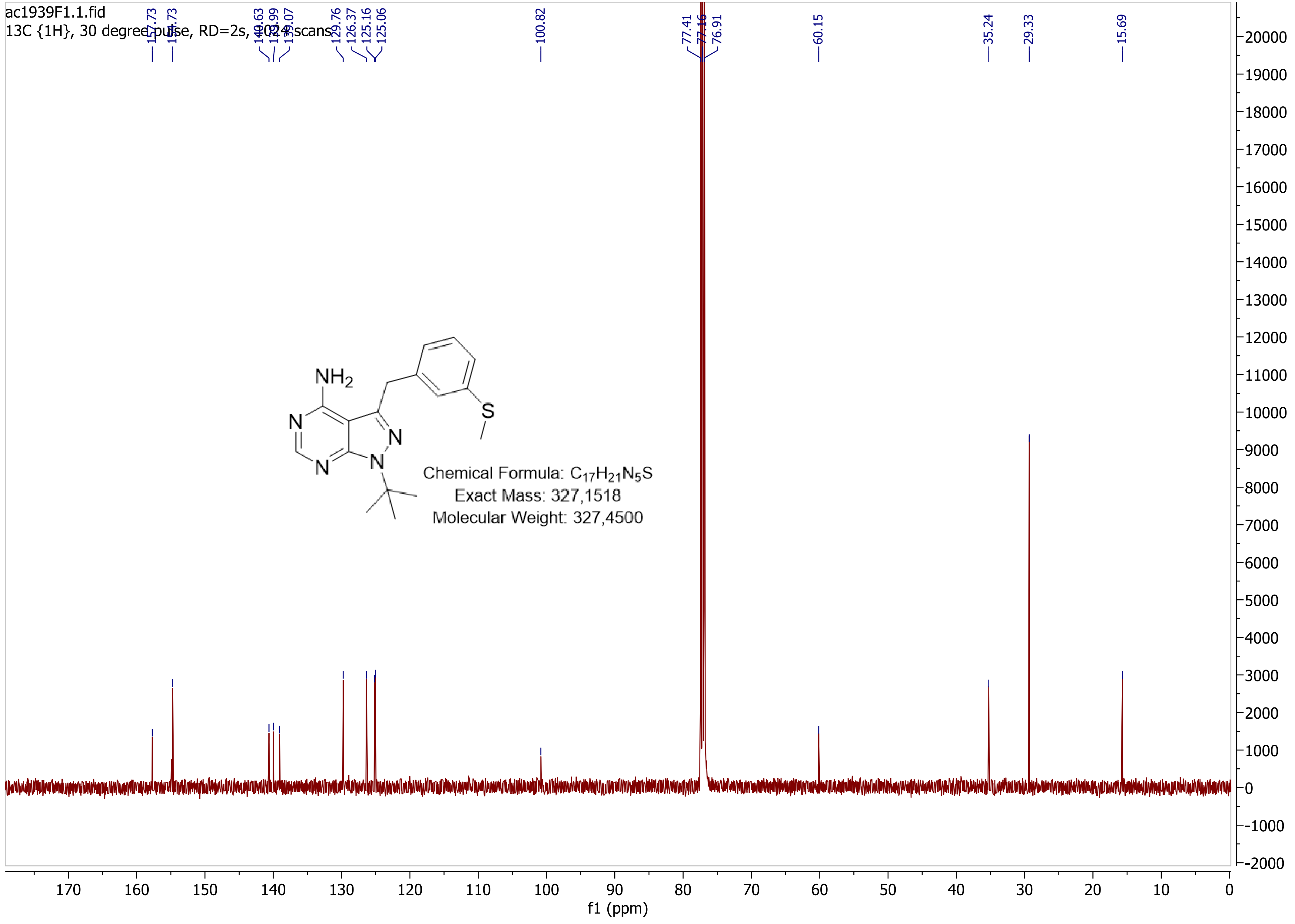

ac1939F1.1.fid
13C {1H}, 30 degree pulse, RD=2s, 1024 scans
157.73
154.73
140.63
139.99
139.07
129.76
126.37
125.16
125.06
100.82
77.41
77.16
76.91
60.15
35.24
29.33
15.69
NH2
N
N
N
N
S
Chemical Formula: C17H21N5S
Exact Mass: 327,1518
Molecular Weight: 327,4500
f1 (ppm)
